# Variation in infection prevention practices for peripherally inserted central venous catheters: A survey of neonatal units in England and Wales

**DOI:** 10.1371/journal.pone.0204894

**Published:** 2018-11-01

**Authors:** Caroline Fraser, Katie Harron, Laura Dalton, Ruth Gilbert, Sam J. Oddie, on behalf of the PREVAIL Study

**Affiliations:** 1 UCL Great Ormond Street Institute of Child Health, London, United Kingdom; 2 Bradford Neonatology, Bradford Royal Infirmary, Bradford, United Kingdom; 3 Centre for reviews and dissemination, University of York, York, United Kingdom; Centre Hospitalier Universitaire Vaudois, FRANCE

## Abstract

**Background:**

There is uncertainty about the variation in infection prevention practices for central venous catheters (CVC) in neonatal units (NNUs) and how practices relate to national guidance.

**Aim:**

To evaluate evidence supporting infection prevention practices for CVCs recommended in national guidelines and to compare with reported practices for peripherally inserted central catheters (PICC), a type of CVC widely used in NNUs.

**Design:**

We searched national guidelines for neonates and children to identify infection prevention practices for CVCs and conducted an overview of studies to determine the quality of evidence underpinning recommendations. We surveyed 134 NNUs in England and Wales to ascertain reported practice.

**Results:**

We found low quality evidence supporting CVC care bundles and use of 2% alcoholic chlorhexidine to decontaminate catheter ports and skin before insertion. Moderate quality evidence supported recommendations against routinely replacing CVCs and against chlorhexidine-impregnated dressings. 90% (44/49) of NICUs and 40% (34/85) of LNUs responded. 66% (48/73) of NNUs reported using CVC care bundles for insertion; 62% (45/73) used bundles for maintenance. 63% (32/51) of those using bundles reported monitoring adherence. 85% (61/72) of NNUs did not routinely replace PICCs and 89% (63/71) did not use chlorhexidine-impregnated dressings. Antiseptic use varied with alcoholic 2% chlorhexidine used for skin preparation in 33% (23/71) of NNUs and for catheter ports in 52% (37/71).

**Conclusions:**

Lack of consistency across NNUs in antiseptic use and low rates of reported CVC care bundle use may reflect the low quality of evidence of the effectiveness and safety of these interventions in NNUs. Clinical trials are needed to quantify benefits and harms of infection prevention practices in NNUs.

## Introduction

Central venous catheters (CVCs) allow the administration of parenteral nutrition, fluids and medication in neonatal units (NNUs). Peripherally inserted central catheters (PICCs) are a type of CVC often used in NNUs because they can be inserted at the bedside and can remain in situ for many days. However, CVCs increase the risk of bloodstream infection (BSI).[1, 2] Neonatal BSI is associated with mortality, increased length of stay and long term morbidity.[3–5] Pathogens colonising the skin enter the bloodstream during CVC insertion or through migration along the catheter surface after insertion.[6–8] In addition, pathogens may be introduced into the blood stream when the CVC connection is breached to administer medication or fluids.[7, 9] Neonates have an elevated risk of BSI compared with older children and adults due to their immunological immaturity, thinner and more permeable skin, exposure to frequent invasive procedures and reliance on parenteral nutrition.[10–12]

In the UK, consensus guidelines for CVC care that reduce the risk of BSI are based mainly on evidence in adults.[13] Guidelines are needed that specifically address CVC practices for neonates as adverse effects can occur in neonates when using alcohol, chlorhexidine and iodine based skin antiseptic solutions.[14–17] CVC replacement is difficult in neonates, guidance is needed on how to minimise infection risk while maintaining the CVC in situ.[18]

In this study, we conducted an overview of national and international guidance, systematic reviews, randomised controlled trials (RCTs) and observational studies to determine the strength of evidence supporting infection prevention practices for CVCs in neonates. We also surveyed NNUs in England and Wales to determine reported adoption of these practices. We hypothesised that variation in adoption of PICC infection prevention practices would be greatest for those practices with the lowest quality supporting evidence or where evidence is uncertain or suggests the practice is potentially harmful for neonates.

## Materials and methods

### Evaluation of evidence and guidelines

Five CVC infection prevention practices relevant to NNU practice and likely to be measurable by a short survey were selected from the EPIC3 UK guidelines by two neonatologists (SO, LD) (Table 1).[13] We searched PubMed for systematic reviews of studies that evaluated CVC related infection prevention practices in NNUs and PICUs, details of search are given in S1 appendix A. Although our focus was on NNUs, more than half of PICU admissions are aged less than 12 months old and we expect practice for infants and neonates to be similar. We additionally searched for studies published since the latest review for each practice (S1 File). We also included any other relevant studies by searching citations and related articles of included studies. We used the GRADE approach to evaluate the evidence supporting each practice in neonates.[19] GRADE is a system for rating quality of evidence and strength of recommendations that initially grades RCTs as high quality evidence and observational studies as low quality evidence. Evidence is graded up or down based on study limitations, inconsistency of results, indirectness of evidence, imprecision, reporting bias, and the magnitude of the treatment effect.

**Table 1:** Recommended practice, supporting evidence and quality and strength of evidence according to GRADE criteria.

Practice	EPIC3 guideline recommendation	Supporting evidence (summary of studies in S1 File)	Quality and strength of evidence using GRADE
**1) Use of a care bundle for (a) insertion or (b) maintenance of PICCs and (c) monitoring compliance of care bundles**	Use quality improvement interventions including protocols for catheter insertion and maintenance, audit of compliance with practice and feedback to practitioners. Guidelines note that these are commonly implemented as bundles.	A systematic review of CVC bundles in all ages and three reviews of CVC bundles in neonates or children found evidence of a reduction in BSI associated with CVC bundles, but the included studies were of low quality.[24–27] The only RCT identified evaluated a parenteral nutrition care bundle in neonates and found no difference in rates of late onset sepsis between the bundle and standard care.*[28]* We found six subsequent ‘before versus after’ studies, all of which reported reduced BSI rates after introduction of CVC bundles.[29–34] Only one study (KH) took into account pre-existing BSI trends and adjusted for case mix.[32] This UK study found a small but significant (14%) reduction in BSI rate after introduction of CVC bundles. CVC bundles were consistently associated with a reduction in BSI rates in before vs after studies.	Low quality of the evidence, weak strength of recommendation (observational studies, one RCT found no difference)
**2) Routine replacement or removal of PICCs after a specified time period**	Do not routinely replace CVCs to prevent infections.	We found no systematic reviews that evaluated routine replacement of CVCs in neonates or children. One systematic review of 12 RCTs in adults concluded that routine replacement of CVCs does not reduce the rate of BSI compared with replacement as needed.[35]	Moderate quality evidence, strong strength of recommendation (RCT evidence in adults is indirect)
**3) Use of chlorhexidine-impregnated foam dressing at site of PICC insertion.**	Do not use chlorhexidine-impregnated foam dressing in neonates. Only use in adults.	A systematic review of RCTs evaluating the efficacy and safety of antimicrobial dressings in reducing BSI for neonates only identified one small RCT evaluating chlorhexidine-impregnated dressings.[36] Chlorhexidine-impregnated dressings have a moderate effect on catheter colonisation, no significant effect on BSI and a high risk of contact dermatitis.	Moderate quality evidence, strong strength of recommendation (RCT is small and subject to bias but shows risk of harm with no effect on BSI)
**4) 2% chlorhexidine in alcohol for skin preparation prior to insertion of PICC.**	Apply 2% chlorhexidine in alcohol at the CVC insertion site.	Two reviews, conducted 10 years apart, found no RCTs that showed a significant benefit of chlorhexidine for skin preparatin in neonates on rates of BSI.[37, 38] There are concerns regarding the toxicity of chlorhexidine and alcohol, particularly neurotoxicity in the case of chlorhexidine.[39–42]) A recent RCT in neonates found 10% providone-iodine and 2% chloehexidine in alcohol to be equally effective at reducing CLABSI with no difference in skin damage, but the study was underpowered.[43]	Low quality evidence, weak strength of recommendation (one underpowered RCT and evidence of harm)
**5) Cleaning of catheter ports with 2% chlorhexidine in alcohol.**	Decontaminate CVC access ports with 2% chlorhexidine in alcohol.	No studies met our inclusion criteria. One before and after study in neonates that did not report pre-existing trends found a reduction in BSI when changing from 70% isopropyl alcohol alone to 2% chlorhexidine in 70% isopropyl alcohol to clean CVC connectors.[44] The evidence in adults cited in the epic3 guidelines should be applicable to neonates as the mechanism for colonisation is unlikely to differ in the two age groups.[45, 46]	Low quality evidence, weak strength of recommendation (no RCTs in correct population)

### Study population and survey design

We developed a short questionnaire about PICC care practices to be completed by neonatologists or senior neonatal nursing staff in NNUs in England and Wales (S2 File). Questions about antiseptic agents in NNUs referred to typical practice for a 29 week gestation baby weighing 900g and provided a list of antiseptics for respondents to choose from. Other questions gave the options yes, no or not applicable. Questions on cleaning of the CVC extension set included photographs of relevant parts of the PICC. We encouraged respondents to answer the survey in collaboration with colleagues so answers reflected agreed practice at the NNU.

After piloting the survey at four neonatal intensive care units (NICUs), the original survey contained 23 questions. We distributed the survey to a consultant contact at each of 134 NNUs: 49 NICUs which provide care for the most unwell or unstable babies and 85 local neonatal units (LNUs) which provide high dependency care to babies who require highly skilled staff but require a lower staff to patient ratios.[20] Special care baby units (SCBUs) for babies who require neonatal care other than intensive or high dependency care were not included in the study as PICCs are not usually used in these units. Contacts were identified from the National Neonatal Audit Programme contact list for all neonatal units in the UK.[21] The survey was first distributed in December 2015 using the online survey platform SurveyMonkey with the option to print the survey and return by post or email. Between December 2015 and May 2016, we sent five follow up emails containing the original online survey to the consultant contacts and advanced neonatal nurse practitioners identified from the Neonatal Nurses Association contact list.

To improve the response rate, we condensed the survey into a printable 2-page document containing 10 questions, prioritising questions judged clinically important, previously well completed, describing measurable practices or covered by existing systematic reviews. In May 2016 we re-sent the survey to NNUs that had not yet provided a complete response. In June 2016, we sent a personalised final email reminder to complete the condensed survey non-responding NICUs, which was addressed to a consultant known at the unit by the clinical investigator for the study (SO). NICUs were prioritised as they use PICCs most frequently.

### Analysis

We counted responses from NNUs with no questions answered as non-respondents, but responses that answered at least one question were included. Where we received multiple responses from the same NNU, we included answers from the first responding consultant in the main analysis. An additional duplicate analysis compared multiple responses from the same NNU to assess consistency of answers. We classified multiple responses as in agreement if more than one professional from a NNU provided the same answer.

### Ethics

This study did not require Research Ethics Approval as we asked staff about their usual practice.[22] Informed consent for completion of the survey was obtained by virtue of completion and return of the survey in accordance with Health Research Authority guidance.[23] Additional consent to acknowledge participants by name was gained by participants ticking a box.

## Results

### Guidelines and evidence

The practices selected for our study were: use of CVC care bundles for insertion and maintenance, monitoring of bundle compliance, use of chlorhexidine impregnated dressing, routine removal or replacement of CVCs, and use of 2% chlorhexidine in alcohol for skin preparation and port disinfection.

We found low quality evidence supporting the use of care bundles, and cleaning catheter ports with 2% chlorhexidine in alcohol and moderate quality evidence to support recommendations to avoid use of chlorhexidine impregnated foam dressings and to avoid routine replacement of CVCs (Table 1). The remaining practices were only supported by low quality evidence. We have combined evidence for use of, and audit and compliance to bundles because most bundles studied include a measure of compliance and we did not identify any studies that assess the efficacy of measuring compliance independently from use of a bundle.

### Responses

We obtained at least one response to the original survey from 28% (24/85) of LNUs and 55% (27/49) of NICUs in England and Wales (S1 Fig). Initially, responses were returned from 23% (5/22) of NICUs that received the condensed questionnaire, but following personalised e-mails to non-responding NICUs the response rate increased to 77% (17/22). Overall 58% (78/135) of NNUs provided at least one response, from 90% (44/49) NICUs and 40% (34/85) LNUs. The condensed, two-page questionnaire (15 responses) was mostly completed on screen (12) with 3 respondents returning a printed survey. Full responses are provided in S3 File.

### Duplicate analysis

Forty eight responses from 21 units were analysed in the duplicate analysis. Duplicate respondents from the same NNU gave consistent answers for questions concerning use of bundles, monitoring compliance of bundles and routine replacement or removal of PICCs (Table 2). However, duplicate respondents often gave divergent responses to questions about antiseptic agents for skin preparation and catheter port cleaning.

**Table 2:** Comparison of reported practices from NNUs that provided duplicate responses.

Practice	NNUs that provided the same answer in duplicate responses*
**1a) Use a care bundle for PICC insertion**	14/14 (100%)
**1b) Use a care bundle for PICC maintenance**	12/14 (86%)
**1c) Monitor and feedback bundle compliance**	12/14 (86%)
**2) No routine removal or replacement of PICCs**	13/15 (87%)
**3) Avoid CHX-impregnated foam dressing**	9/14 (64%)
**4) Use 2% CHX in alcohol as skin preparation**	6/13 (46%)
**5) Clean catheter ports with 2% CHX in alcohol**	7/14 (50%)

*21 NNUs had more than one response to at least one question. We show the number of NNUs with more than one response for each question. CHX = chlorhexidine

### Reported practice

Two thirds of NNUs reported using care bundles for PICC insertion and maintenance (Table 3). There were five NNUs that reported using only one type of bundle. The majority of NNUs reported that PICC replacement was not routine and did not use chlorhexidine impregnated foam dressings. Of the 11 NNUs that reported routine removal or replacement, ten gave the time at which they would remove or replace the catheter and the remaining NNU was in process of devising a protocol. The median number of days to removal or replacement was 14 (range 7 to 42). Four of those that reported routine removal or replacement commented that the limit could be extended if necessary.

**Table 3:** Reported practice stratified by unit level.

Practice	Number of units that report each practice					
	All NNUs (n = 77)		NICUs (n = 44)		LNUs (n = 34)	
**1a) Use a care bundle for PICC insertion**	66%	(48/73)	72%	(31/43)	57%	(17/30)
**1b) Use a care bundle for PICC maintenance**	62%	(45/73)	65%	(28/43)	57%	(17/30)
**1c) Monitor and feedback bundle compliance**	63%	(32/51)	66%	(21/32)	58%	(11/19)
**2) No routine removal or replacement of PICCs**	85%	(61/72)	79%	(34/43)	93%	(27/29)
**3) Avoid CHX-impregnated foam dressing**	89%	(63/71)	93%	(39/42)	83%	(24/29)
4) Use 2% CHX in alcohol as skin preparation*	33%	(23/71)	41%	(17/42)	21%	(6/29)
**5) Clean catheter ports with 2% CHX in alcohol**	52%	(37/71)	55%	(23/42)	48%	(14/29)

NICUs = neonatal intensive care units, LNU = high dependency care unit, CHX = chlorhexidine *For a 29 week gestation baby weighing 900g

**Fig 1:**
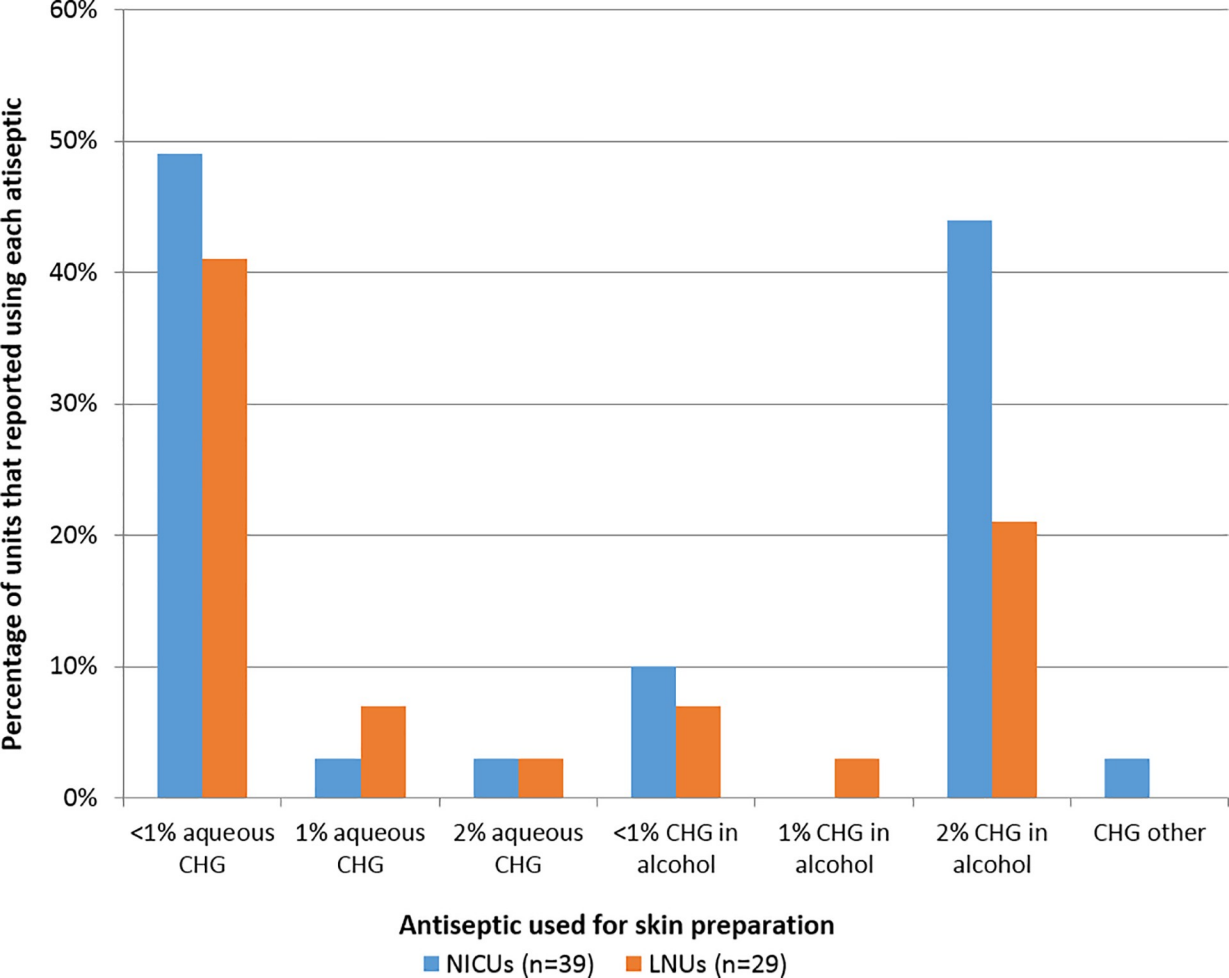
Antiseptics that were reported for skin preparation prior to CVC insertion. NICUs = neonatal intensive care units, LNUs = high dependency care units; CHX = chlorhexidine; units could select multiple choices therefore the sum of the percentages may be greater than 100.

All NNUs reported using a form of chlorhexidine for skin disinfection prior to insertion but the concentration and use of alcohol vs aqueous solutions varied with one-third using 2% chlorhexidine in alcohol (Fig 1). Ten respondents reported a two-step technique for skin antisepsis prior to PICC insertion, for example applying 1% aqueous chlorhexidine initially and performing a second clean with 2% chlorhexidine in alcohol.

**Fig 2:**
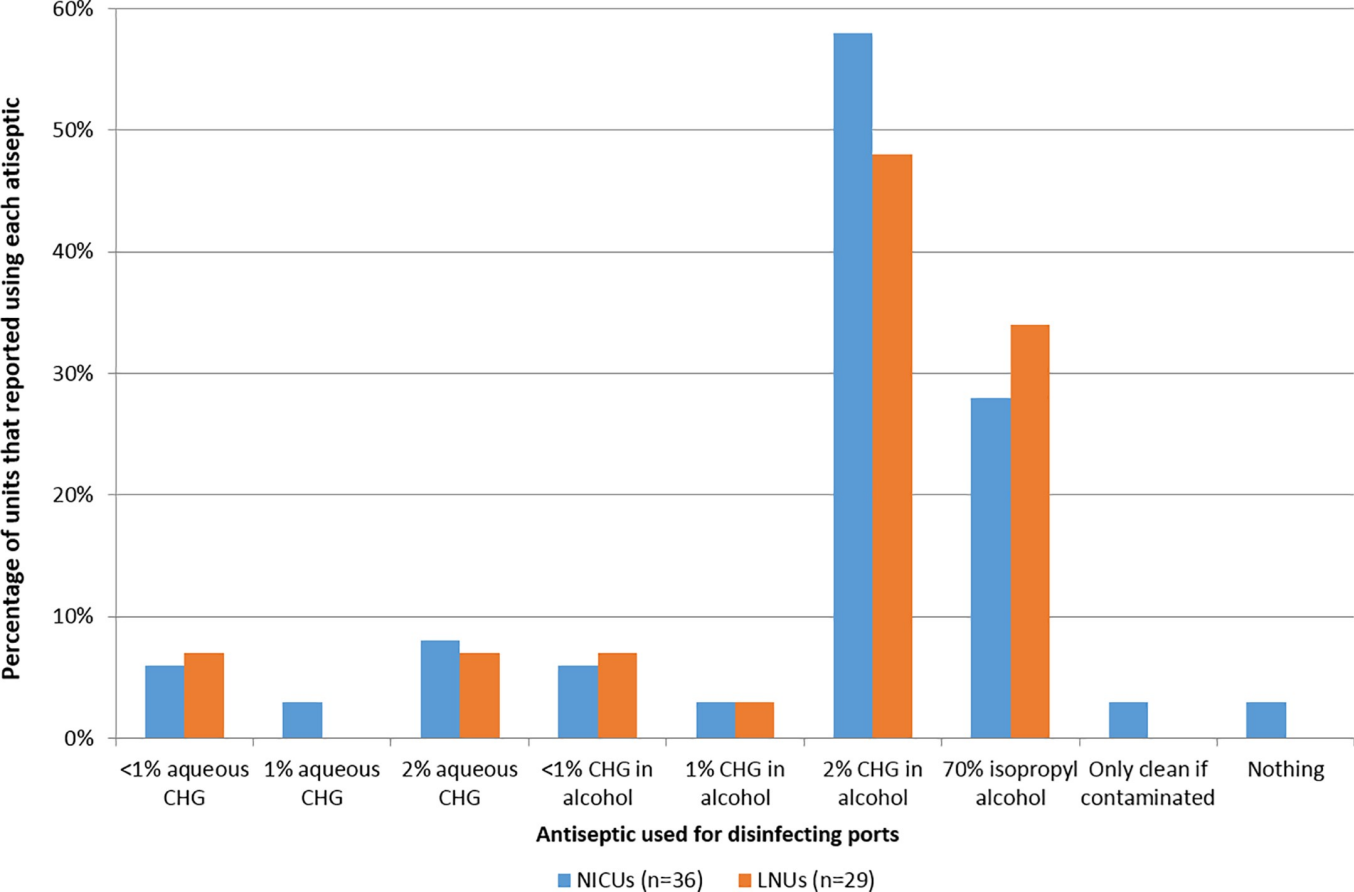
Reported antiseptic use for disinfection of catheter ports. NICUs = neonatal intensive care units, LNUs = high dependency care units; CHX = chlorhexidine; units could select multiple choices therefore the sum of the percentages may be greater than 100.

2% chlorhexidine in alcohol was the most commonly used antiseptic for cleaning catheter ports, followed by 70% isopropyl alcohol (Fig 2). Nine respondents reported having a two-step process for catheter port cleaning.

There was no difference in use of 2% chlorhexidine in alcohol to clean catheter ports between NNUs that use bundles and those that did not, but use of 2% chlorhexidine in alcohol as skin preparation was more common in bundle users than non-users (Table 4).

**Table 4:** Reported antiseptic use in NNUs that used bundles and those that did not for NNUs that responded to both questions.

Practice	Bundle users (n = 48)	Bundle non-users (n = 21)
**4) Use 2% CHX in alcohol as skin preparation**	19 (40%)	4 (19%)
**5) Clean catheter ports with 2% CHX in alcohol**	25 (52%)	11 (52%)

CHX = chlorhexidine

## Discussion

We found variation in adherence to recommended care practices, and a lack of high quality evidence supporting these in neonates. Reported practice from the majority of NNUs was in accordance with recommendations against routine replacement of PICCs and against use of chlorhexidine-impregnated foam dressing, supported by moderate quality evidence. Despite only low quality evidence to support the use of care bundles in neonates, the majority of NNUs reported using insertion and maintenance bundles.

A strength of the study was the response rate of 88% for NICUs. The lower response rate from LNUs may reflect fewer PICC insertions in LNUs or a higher level research engagement among NICU practitioners. The high response rate from NICUs was achieved after reducing the questionnaire length and repeated personal reminders. Other studies have highlighted the importance of personal contact.[47] A limitation of the study is that we analysed reported practice, rather than actual practice. The duplicate analysis demonstrates the validity for the questions on use of care bundles and routine removal or replacement of PICCs. However, the inconsistencies for chlorhexidine dressing and antiseptic use suggest there is uncertainty regarding these topics. It is unclear whether the uncertainty is related to the survey design, for example respondents misunderstanding the question or accidentally selecting the wrong answer, or due to real differences in practice within units.

Variation in chlorhexidine solutions may reflect differing interpretations of case studies that report burns for aqueous and alcoholic chlorhexidine in very preterm babies.[15, 16, 39, 41, 42, 48, 49] Many before and after studies have reported statistically significant reductions in BSI rates following adoption of care bundles.[27] The volume and consistency of these studies is likely to impact practice and opinion despite their low quality and risk of bias, and is a potential reason for the greater adoption of these practices in contrast to use of 2% chlorhexidine in alcohol. Variation in antiseptic solution used to clean skin before CVC insertion has been reported elsewhere. Surveys of UK NICUs found 2% chlorhexidine in alcohol used in no units in 2008 but in 39% (22/57) of units in 2013.[50, 51] This suggests the use of 2% chlorhexidine in alcohol is a recent development in NNUs, which may follow the Matching Michigan initiative.[52]

Use of 2% chlorhexidine in alcohol was more common in NNUs that used care bundles than those that did not. This practice is included in the Department of Health Saving Lives care bundle and the NHS England Matching Michigan programme, both of which were evidence based interventions to reduce BSI rates in adult and paediatric intensive care.[52, 53] There have been no equivalent national initiatives for neonatal intensive care. This suggests some NNUs use bundles for adults and older children. There was less consistency between units for use of care bundles and antiseptics compared to avoidance of routine replacement of CVCs and chlorhexidine dressing. Factors influencing adoption of practices will include the underlying evidence base, but also the experience of clinicians, organisational drivers such as the UK’s Matching Michigan Programme and perceptions as to whether practices have likely harms associated with them.

Practices with small effect sizes may have a greater value to neonates compared to other populations due to the higher rate of BSI in NNU, therefore all practices would benefit from neonate specific evidence.[54, 55] Antiseptics have the most urgent need to be studied in neonates due to the increased risk of adverse events. Without high quality evidence, there may be a role for guidelines on a small scale to harmonise local practice and facilitate audit and quality improvement. However, use of guidelines that are based on insufficient evidence on a large scale, without due consideration of the quality of underpinning evidence, runs the risk of encouraging clinicians to carry out practices that are either without value, or even harmful.

Further research is needed to determine the effectiveness and safety of CVC care practices for reducing BSI in neonates. A multicentre UK RCT evaluating antimicrobial impregnated versus standard PICCs for preventing BSI in preterm neonates is in progress (www.prevail.org.uk). Our findings of inconsistent practice underline the need for adequately powered RCTs to determine the effectiveness and safety of skin antiseptic agents.

## Supporting information

S1 Fig Flow diagram of responses to each stage of the survey. (TIF)

S1 File Appendix A: Literature search. (DOCX)

S2 File Appendix B: Survey. (DOCX)

S3 File Dataset: Survey responses. (XLSX)
