# Supplementary figures and images for: Variation in infection prevention practices for peripherally inserted central venous catheters: A survey of neonatal units in England and Wales
Source: PLoS One. 2018 Nov 1;13(11):e0204894. doi: 10.1371/journal.pone.0204894 (PMC6211675; doi:10.1371/journal.pone.0204894)

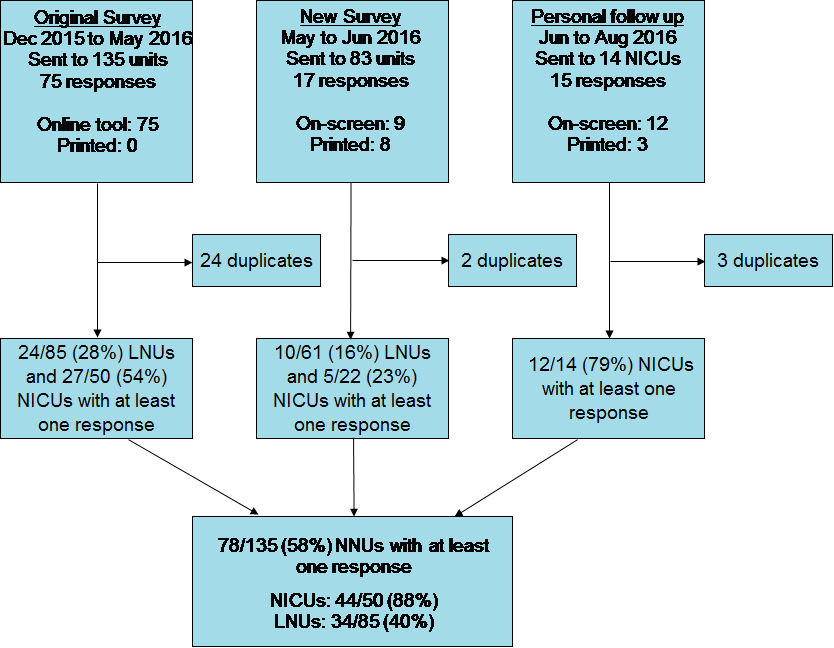

Supplement: S1 Fig — (TIF) [file pone.0204894.s001.tif]
