# Supplementary material for: Variation in infection prevention practices for peripherally inserted central venous catheters: A survey of neonatal units in England and Wales
Source: PLoS One. 2018 Nov 1;13(11):e0204894. doi: 10.1371/journal.pone.0204894 (PMC6211675; doi:10.1371/journal.pone.0204894)
Supplement: S1 File — (DOCX) [file pone.0204894.s002.docx]

**S1 APPENDIX A**

Which infection control practices are effective in reducing BSI in babies with CVCs in NNUs?

**Search 1**

*Aim*

To establish which CVC related infection prevention practices had been the subject of systematic reviews in NNUs or PICUs.

*Search Strategy*

The following search terms were used to search PubMed on 21^st^ February 2017, with no restrictions on date or language:

systematic[sb] AND ((neonate OR baby OR infant OR newborn OR new born OR children OR child OR nicu OR nicus OR picus OR picu OR paediatric OR pediatric OR neonatal OR premature) ((((blood OR bloodstream OR nosocomial OR (health-care OR hospital) acquired) OR line OR catheter OR cvc) (infection)) OR sepsis OR bacteraemia OR clabsi OR bsi OR hcai) (catheter OR cvc OR picc OR line) (control OR strategy OR prevent* OR initiative OR framework OR program* OR protocol OR quality OR improv* OR reduc* OR change OR changes OR changing OR care OR practice))

Returned studies were assessed to see if they met the following inclusion criteria:

- Systematic review
- Evaluates efficacy of a CVC related practice to prevent BSI or CLABSI
- In NNU or PICU

*Results*

The search returned 3492 studies, of which 19 met the inclusion criteria. These were categorised by practice (table 1).

Table 1: Practices identified in literature search

| **Practice** | **Reviews** | **Epic3 recommendation** | **Reason for (non) inclusion** |
| --- | --- | --- | --- |
| Lock solution | 3(1-3) | “Antimicrobial lock solutions should not be used routinely to prevent catheter-related bloodstream infections.” | We did not include antibiotic lock because we believed this was not a common practice in the UK |
| Dressing | 1(4) | “Consider the use of a chlorhexidine-impregnated sponge dressing in adult patients with a central venous catheter as a strategy to reduce catheter-related bloodstream infection.” New recommendation | We decided to focus on chlorhexidine-impregnated dressings as there are safety concerns and it is one of the few recommendations in epic3 that acknowledges a difference between adults and younger patients. |
| Bundles | 4(5-8) | “Use quality improvement interventions to support the appropriate use and management of intravascular access devices (central and peripheral venous catheters) and ensure their timely removal. These may include: protocols for device insertion and maintenance; reminders to review the continuing use or prompt the removal of intravascular devices; audit and feedback of compliance with practice guidelines; and continuing professional education.” | We chose to include a question on bundles as there were several reviews and it had been a focus following the Saving Lives initiative and following Matching Michigan, so we were interested to see how much they were used. |
| Heparin flushing | 4 (9-12) | “Do not use systemic anticoagulants routinely to prevent catheter-related bloodstream infection.” | This is not a common practice in UK neonatal units, due to risk of causing bleeding in fragile premature neonates |
| Impregnated/bonded catheter | 4(13-16) | “Use an antimicrobial-impregnated central venous access device for adult patients whose central venous catheter is expected to remain in place for >5 days if catheter-related bloodstream infection rates remain above the locally agreed benchmark, despite the implementation of a comprehensive strategy to reduce catheter-related bloodstream infection.” | We know from the manufacturer that these are not sold in UK neonatal units. |
| Insertion site antisepsis | 1(17) | “Decontaminate the skin at the insertion site with a single-use application of 2% chlorhexidine gluconate in 70% isopropyl alcohol (or povidone iodine in alcohol for patients with sensitivity to chlorhexidine) and allow to dry prior to the insertion of a central venous access device.” | Interesting to look at as we only found one (old) review in neonates and there is a concern regarding safety |
| Prophylactic antibiotics | 1(18) | “Do not routinely administer intranasal or systemic antimicrobials before insertion or during the use of an intravascular device to prevent catheter colonisation or bloodstream infection.” | Many neonates receive antibiotics so hard to determine which ones would be classed as prophylactic |
| Catheter placement | 1 | “In selecting an appropriate intravascular insertion site, assess the risks for infection against the risks of mechanical complications and patient comfort.”  “Use the upper extremity for nontunnelled catheter placement unless medically contraindicated.” | Calls for clinical judgement, not a standard practice therefore hard to measure in a survey. |
| Routine removal or replacement | 0 | “Do not routinely replace central venous access devices to prevent catheter-related infection.” | Neonates have CVCs in place for longer than adults so there may be a difference in replacement, but no reviews identified… |
| Catheter port cleaning | 0 | “A single-use application of 2% chlorhexidine gluconate in 70% isopropyl alcohol (or povidone iodine in alcohol for patients with sensitivity to chlorhexidine) should be used to decontaminate the access port or catheter hub. The hub should be cleaned for a minimum of 15 s and allowed to dry before accessing the system.” | We decided to include this as we found no evidence in neonates but it was recommended in epic3 |

**Search 2**

*Aim*

To establish the efficacy of CVC care bundles at reducing BSI in NNUs and PICUs

*Search Strategy*

We searched for studies published since the search date of the latest review

The following search terms were used to search PubMed for studies published between 30^th^ June 2015 and 22^nd^ February 2017:

(Neonate OR baby OR infant OR newborn OR new born OR child OR nicu OR nicus OR picus OR picu OR paediatric OR pediatric OR neonatal OR premature) ((((blood OR bloodstream) (infection)) OR sepsis OR bacteraemia OR clabsi OR bsi) (catheter OR cvc OR picc OR line) (control OR strategy OR prevent* OR initiative OR framework OR program* OR bundle OR protocol)

Returned studies were assessed to see if they met the following inclusion criteria:

- One of the following study designs
  - Systematic review of RCTs/cluster RCTs/controlled time series
  - RCT
  - Cluster RCT (accounting for case mix and pre-existing trends)
  - Controlled time series (accounting for case mix and pre-existing trends)
- In a NNU or PICU setting
  - or a review that includes NICU/PICU studies
- Intervention = care bundle
  - No other interventions implemented at same time
  - Bundle is defined as a group of evidence based interventions that are implemented together
- Comparison made to standard care (i.e. no bundle)
- Outcome is BSI or CLABSI
  - Although BSI would be a preferred outcome, nearly all studies looking at bundles use CLABSI as the outcome
  - Laboratory confirmed

*Results*

The search returned 103 studies, however none met our inclusion criteria.

**Search 3**

*Aim*

To establish the efficacy of routine replacement or removal of CVCs at reducing BSI in NNUs and PICUs

*Search Strategy*

The following search terms were used to search PubMed for studies published between 30^th^ June 2015 and 1^st^ March 2017:

(Neonate OR baby OR infant OR newborn OR new born OR child OR nicu OR nicus OR picus OR picu OR paediatric OR pediatric OR neonatal OR premature OR) ((((blood OR bloodstream) (infection)) OR sepsis OR bacteraemia OR clabsi OR bsi) (catheter OR cvc OR picc OR line) (replace* OR remov*)

Returned studies were assessed to see if they met the following inclusion criteria:

Inclusion criteria

- One of the following study designs
  - Systematic review of RCTs
  - RCT
- In a NNU or PICU setting
  - or a review that includes NICU/PICU studies
- Intervention = routine replacement or removal after specified time period
  - No other interventions implemented at same time
- Comparison made to standard care (i.e. not removing until clinically indicated)
- Outcome is BSI or CLABSI
  - Although BSI would be a preferred outcome, nearly all studies looking at bundles use CLABSI as the outcome
  - Laboratory confirmed

*Results*

The search returned 335 studies, of which none met the inclusion criteria.

**Search 4**

*Aim*

To establish the efficacy of chlorhexidine-impregnated dressings at reducing BSI in NNUs and PICUs

*Search Strategy*

We searched for studies published since the search date of the latest review

The following search terms were used to search PubMed for studies published between 1^st^ September 2015 and ?? February 2017:

(Neonate OR baby OR infant OR newborn OR new born OR child OR nicu OR nicus OR picus OR picu OR paediatric OR pediatric OR neonatal OR premature) ((((blood OR bloodstream) (infection)) OR sepsis OR bacteraemia OR clabsi OR bsi) (catheter OR cvc OR picc OR line) (chlorhexidine OR chg OR antisepsis OR antisep* OR antimicrobial) (dressing OR patch)

Returned studies were assessed to see if they met the following inclusion criteria:

- One of the following study designs
  - Systematic review of RCTs/cluster RCTs/controlled time series
  - RCT
- In a NNU or PICU setting
  - or a review that includes NICU/PICU studies
- Intervention = chlorhexidine dressing OR skin preparation OR hub cleaning
  - 2% chlorhexidine in alcohol for skin prep and hub cleaning
  - No other interventions implemented at same time
- Comparison made to standard care (un-impregnated dressing)
- Outcome is BSI or CLABSI
  - Although BSI would be a preferred outcome, nearly all studies looking at bundles use CLABSI as the outcome
  - Laboratory confirmed

*Results*

The search returned 24 studies, however the only studies that met the inclusion criteria were reviews identified in search 1.

**Search 5**

*Aim*

To establish the efficacy of using 2% chlorhexidine as skin preparation prior to insertion at reducing BSI in NNUs and PICUs

*Search Strategy*

There was no search date included in the review we identified for this practice, therefore we searched from two years prior to the publication date.

The following search terms were used to search PubMed for studies published between 1^st^ January 2002 to 3^rd^ March 2017:

(Neonate OR baby OR infant OR newborn OR new born OR child OR nicu OR nicus OR picus OR picu OR paediatric OR pediatric OR neonatal OR premature) ((((blood OR bloodstream) (infection)) OR sepsis OR bacteraemia OR clabsi OR bsi) (catheter OR cvc OR picc OR line) (chlorhexidine OR chg OR antisep* OR disinfect*)

Returned studies were assessed to see if they met the following inclusion criteria:

- One of the following study designs
  - Systematic review of RCTs/cluster RCTs/controlled time series
  - RCT
- In a NNU or PICU setting
  - or a review that includes NICU/PICU studies
- Intervention = chlorhexidine dressing OR skin preparation OR hub cleaning
  - 2% chlorhexidine in alcohol for skin prep and hub cleaning
  - No other interventions implemented at same time
- Comparison made to standard care or another antiseptic
- Outcome is BSI or CLABSI
  - Although BSI would be a preferred outcome, nearly all studies looking at bundles use CLABSI as the outcome

*Results*

The search returned 87 studies, of which one met the inclusion criteria (19).

**Search 6**

*Aim*

To establish the efficacy of using 2% chlorhexidine to clean catheter ports at reducing BSI in NNUs and PICUs

*Search Strategy*

(Neonate OR baby OR infant OR newborn OR new born OR child OR nicu OR nicus OR picus OR picu OR paediatric OR pediatric OR neonatal OR premature) ((((blood OR bloodstream) (infection)) OR sepsis OR bacteraemia OR clabsi OR bsi) (catheter OR cvc OR picc OR line) (chlorhexidine OR chg OR antisep* OR disinfect*) (hub OR port OR access OR connect* OR parenteral nutrition OR pn)

Returned studies were assessed to see if they met the following inclusion criteria:

- One of the following study designs
  - Systematic review of RCTs/cluster RCTs/controlled time series
  - RCT
  - Cluster RCT (accounting for case mix and pre-existing trends)
  - Controlled time series (accounting for case mix and pre-existing trends)
- In a NNU or PICU setting
  - or a review that includes NICU/PICU studies
- Intervention = catheter port cleaning
  - 2% chlorhexidine in alcohol for hub cleaning
  - No other interventions implemented at same time
- Comparison made to standard care or another antiseptic
- Outcome is BSI or CLABSI
  - Although BSI would be a preferred outcome, nearly all studies looking at bundles use CLABSI as the outcome

*Results*

We identified 34 studies, none of which met our inclusion criteria.

Table 2: Description of included studies

| **Author (Year)** | **Design (Duration)** | **Setting (NICU/PICU)** | **Intervention (and comparison group?)** | **Participants** | **Primary outcome** | **Comments** |
| --- | --- | --- | --- | --- | --- | --- |
| **Bundles** |  |  |  |  |  |  |
| Helder et al  (2013) | Review  1 in PICU (B-A study)  3 in NICUs (all B-A studies) | NICUs and PICUs | Intravenous bundle  None included checklist | Control: 749 Intervention: 736  (from 3 studies, one did not report participants) | BSI per 1000 CVC days  Reduction in all 4 studies |  |
| Ista et al.  (2016) | Review  14 in NICUs (all B-A studies)  14 in PICUs (2 ITS; 12 B-A studies)  8 in mix of PICU/NICU/Adult ICU (all B-A studies) | PICUs and NICUs | Central line bundle  9/14 NICU included checklist  8/14 PICU included checklist  6/8 mixed included checklist | NICUs  Pre: 3163  Intervention: 51 (1 study)  Post: 3807  (from 7 studies, 7 did not report participants)  PICUs  Pre: 3900 (9 studies)  Intervention: 2294 (2 studies)  Post: 5645 (11 studies)  2^nd^ post: 413 (1 study)  (4 studies did not report participants)  Mixed  Pre: 7,830 (2 study)  Post: 46,277 (2 study)  (6 studies did not report participants) | Effect of implementing central line bundle on CLABSI  PICUs  IRR 0.58 (0.48-0.71)  NICUs  IRR 0.47 (0.83-0.59) |  |
| Smulders et al. (2013) | Review  2 studies in NICUs (2 B-A studies)  7 studies in PICUs (2 ITS; 5 B-A studies) | PICUs and NICUs | Central line bundle  5/7 PICU studies include checklist, neither NICU study includes checklist | N/A | CLABSI/1000 line days  Decreased |  |
| Ng et al.  (2015) | B-A Study  Baseline: 10 months  Intervention: 14 months | PICU | Training interns | Baseline: 285  Intervention: 436 | CLABSI/1000 line days  Baseline: 25.2  Intervention: 9.3  BSI/1000 admissions  Baseline: 88  Intervention: 41 | Shows pre-existing trends.  Comparison between baseline and intervention groups show some differences between catheter use. |
| Piazza et al.  (2016) | B-A Study  Baseline: 12 months  Intervention: 12 months | PICU | 16 Centres allocated 8 different combination of components to implement  Included monitoring hub care compliance at some centres | Line days  Baseline: 116,987  Intervention: 119,003 | CLABSI/1000 line days  Baseline: 1.333  Intervention: 1.076 | Group that included hub care monitoring and sterile tube change techniques had greatest decrease  No association between compliance (to hub care monitoring/sterile tube change etc) and CLABSI |
| Rallis | B-A Study  Baseline: 9 months  Intervention: 3 months  Post-intervention: 9 months | NICU | QI measures including education and aseptic technique | Baseline: 94  Post-intervention: 59 | CLABSI/1000 line days  Baseline: 12  Post-intervention: 3.4 | Comparison between baseline and intervention groups shows no significant differences  No pre-existing trends reported |
| Harron et al. | ITS  Pre-implementation: 96 months  Post-Implementation: 24 months | PICUs | CVC bundles | N/A | BSI/1000 bed days  Baseline: 4.47 (95% CI: 2.52 to 6.42)  24 months post-intervention: 3.3 (95% CI: 1.34 to 5.32)  Observed rate fell 26% but trend was already downwards, assuming trends had been consistent would be 3.92 post implementation therefore 15% rate reduction | Not identified in search but we were aware of study (KH author) |
| **Routine replacement** | |  |  |  |  |  |
| Cook  (1997) | Review  12 RCTs in adults | Adults | Guidewire exchange (GWX) vs new site replacement (NSR) | Number of catheters  NSR: 215 (5)  GWX: 348 (5)  NSR regular intervals: 504 (5)  GWX regular intervals: 419 (4)  NSR as needed: 241 (3)  GWX as needed: 163 (2)  GWX at 3 days and NSR every 7 days: 115 (1) | NSR every 3 days vs NSR as needed  Cather-related bacteraemia RR: 0.93 (95% CI: 0.27 to 3.20) | Review in adults identified from guidelines because no studies met criteria  2 trials that compared regular change to as needed most relevant to question |
| **Chlorhexidine dressing** | |  |  |  |  |  |
| Lai et al.  (2016) | Review  1 study on CHG dressing | NICUs | Chlorhexidine dressing vs PI | 655 babies (GA 22.5 to 26.5) | CRBSI/1000 days  RR 1.18 (0.53 to 2.65)  Catheter colonisation/1000  RR 0.62 (0.45 to0.86)  Contact dermatitis/1000  RR 43.06 (2.61 to 710.44) | Other studies in review look at other chlorhexidine studies but not at dressing |
| **Chlorhexidine skin prep** | |  |  |  |  |  |
| Carson et al.  (2004) | Review  8 studies (5 RCTs, 1 quasi-experimental and 1 meta-analysis of 8 RCTs) | Children | PI vs CHG (for preventing CVC realted site infections and bacteraemia) | N/A | CHG superior to PI in most studies for preventing colonisation of insertion site and catheter tip but conflicting evidence for preventing CLABSI | Recommends against use of chlorhexidine in infants born <37 weeks or LBW until further research |
| Ponnusamy et al.  (2014) | Review  4 studies (3 RCTs, 1 retrospective) | NICU | PI vs IPA + CHG  PI vs aq CHG  0.5% aq CHG vs 0.05% aq CHG | N/A | Small RCT demonstrated better bactericidal activity with 0.5% CHG compared to 0.05%  Comparisons of PI to CHG showed no difference in CLABSI but one RCT reported less contamination of blood cultures with CHG |  |
| **Port disinfection** | |  |  |  |  |  |
| Bishay et al.  (2011) | B-A study | NICU – surgical infants receiving parenteral nutrition (PN) | 70% IPA vs 2% chlorhexidine in 70% isopropanol alcohol | Before (alcohol): 98  After (CHG): 112 | Sepsis per 100 days of PN  IRR: 0.72 (95% CI 0.61 to 0.84)  Septicemia per 100 days of PN  IRR: 0.49 (95% CI: 0.36 to 0.67) |  |
| Ruschman and Fulton  (1993) | Experimental | In vitro | 70% IPA vs PI vs control (no disinfectant) |  | 0 CFUs for alcohol  2/5 CFUs for PI  Control as expected |  |
| Casey et al.  (2003) | 1 RCT | Surgical patients | Posiflow needleless connector vs standard cap  CHG in IPA vs alcohol vs 10% aq PI for skin preparation and disinfection of the intravenous connections | Control – 39 participants  PosiFlow – 38 participants  Posiflow  0.5% CHG in alcohol: 91  70% IPA: 91  10% PI: 92  Standard caps  0.5% CHG in alcohol: 102  70% IPA: 102  10% PI: 102 | External Posiflow contamination  0.5% CHG: 31% (28)  70% IPA: 69% (63)  10% PI: 25% (23)  Internal Posiflow contamination  0.5% CHG: 3% (3)  70% IPA: 10% (9)  10% PI: 9% (8)  Port contamination (standard cap)  0.5% CHG: 17% (17)  70% IPA: 22% (22)  10% PI: 16% (16)  Port contamination (Posiflow)  0.5% CHG: 1% (1)  70% IPA: 10% (9)  10% PI: 9% (8) | Both skin preparation and connector disinfection |

B-A study = before versus after study, ITS = interrupted time series, RCT = randomised controlled trial, CLABSI = central line associated bloodstream infection, PICU = paediatric intensive care unit, NICU = neonatal intensive care unit, PI = providone-iodine, CHG = chlorhexidine gluconate, aq=aqueous, IPA = isopropyl alcohol

1. Abu-El-Haija M, Schultz J, Rahhal RM. Effects of 70% ethanol locks on rates of central line infection, thrombosis, breakage, and replacement in pediatric intestinal failure. Journal of pediatric gastroenterology and nutrition. 2014;58(6):703-8.

2. Oliveira C, Nasr A, Brindle M, Wales PW. Ethanol locks to prevent catheter-related bloodstream infections in parenteral nutrition: a meta-analysis. Pediatrics. 2012;129(2):318-29.

3. Taylor JE, Tan K, Lai NM, McDonald SJ. Antibiotic lock for the prevention of catheter-related infection in neonates. The Cochrane database of systematic reviews. 2015(6):Cd010336.

4. Lai NM, Taylor JE, Tan K, Choo YM, Ahmad Kamar A, Muhamad NA. Antimicrobial dressings for the prevention of catheter-related infections in newborn infants with central venous catheters. The Cochrane database of systematic reviews. 2016;3:CD011082.

5. Helder O, van den Hoogen A, de Boer C, van Goudoever J, Verboon-Maciolek M, Kornelisse R. Effectiveness of non-pharmacological interventions for the prevention of bloodstream infections in infants admitted to a neonatal intensive care unit: A systematic review. International journal of nursing studies. 2013;50(6):819-31.

6. Ista E, van der Hoven B, Kornelisse RF, van der Starre C, Vos MC, Boersma E, et al. Effectiveness of insertion and maintenance bundles to prevent central-line-associated bloodstream infections in critically ill patients of all ages: a systematic review and meta-analysis. The Lancet Infectious diseases. 2016;16(6):724-34.

7. Legemaat MM, Jongerden IP, van Rens RM, Zielman M, van den Hoogen A. Effect of a vascular access team on central line-associated bloodstream infections in infants admitted to a neonatal intensive care unit: a systematic review. International journal of nursing studies. 2015;52(5):1003-10.

8. Smulders CA, van Gestel JP, Bos AP. Are central line bundles and ventilator bundles effective in critically ill neonates and children? Intensive care medicine. 2013;39(8):1352-8.

9. Bradford NK, Edwards RM, Chan RJ. Heparin versus 0.9% sodium chloride intermittent flushing for the prevention of occlusion in long term central venous catheters in infants and children: A systematic review. International journal of nursing studies. 2016;59:51-9.

10. Long DA, Coulthard MG. Effect of heparin-bonded central venous catheters on the incidence of catheter-related thrombosis and infection in children and adults. Anaesthesia and intensive care. 2006;34(4):481-4.

11. Shah PS, Ng E, Sinha AK. Heparin for prolonging peripheral intravenous catheter use in neonates. The Cochrane database of systematic reviews. 2005(4):Cd002774.

12. Shah PS, Ng E, Sinha AK. Heparin for prolonging peripheral intravenous catheter use in neonates. The Cochrane database of systematic reviews. 2002(4):Cd002774.

13. Balain M, Oddie SJ, McGuire W. Antimicrobial-impregnated central venous catheters for prevention of catheter-related bloodstream infection in newborn infants. The Cochrane database of systematic reviews. 2015(9):Cd011078.

14. Shah PS, Shah N. Heparin-bonded catheters for prolonging the patency of central venous catheters in children. The Cochrane database of systematic reviews. 2014(2):Cd005983.

15. Shah PS, Shah N. Heparin-bonded catheters for prolonging the patency of central venous catheters in children. The Cochrane database of systematic reviews. 2007(4):Cd005983.

16. Thomas R, Lee S, Patole S, Rao S. Antibiotic-impregnated catheters for the prevention of CSF shunt infections: a systematic review and meta-analysis. British journal of neurosurgery. 2012;26(2):175-84.

17. Carson SM. Chlorhexidine versus povidone-iodine for central venous catheter site care in children. Journal of pediatric nursing. 2004;19(1):74-80.

18. Lodha A, Furlan AD, Whyte H, Moore AM. Prophylactic antibiotics in the prevention of catheter-associated bloodstream bacterial infection in preterm neonates: a systematic review. Journal of perinatology : official journal of the California Perinatal Association. 2008;28(8):526-33.

19. Ponnusamy V, Venkatesh V, Clarke P. Skin antisepsis in the neonate: what should we use? Current opinion in infectious diseases. 2014;27(3):244-50.
