# Supplementary material for: Variation in infection prevention practices for peripherally inserted central venous catheters: A survey of neonatal units in England and Wales
Source: PLoS One. 2018 Nov 1;13(11):e0204894. doi: 10.1371/journal.pone.0204894 (PMC6211675; doi:10.1371/journal.pone.0204894)
Supplement: S2 File — (DOCX) [file pone.0204894.s003.docx]

**Survey of infection control practices for peripherally inserted central catheters (PICC) in neonatal units**

| We are asking you to complete a short survey about the procedures in your unit for preventing catheter infections. We will use the information from the survey together with national data on variation in bloodstream infections in NICUs to estimate the potential benefits of interventions to reduce infections related to central venous catheters. The survey is being carried out by investigators for the PREVAIL randomised trial of antibiotic versus standard PICCs to estimate the benefit to babies across all NICUs in the NHS ([www.prevailtrial.org.uk](http://www.prevailtrial.org.uk)) which is funded by the NIHR.  Please complete all questions and mark answers clearly with a dark pen. If you do not know the answers, please give the email or name and telephone number for someone who can provide the answers. The form is designed to be printed out so that you can discuss the questions with the clinical team. If you prefer, you may enter your responses directly into the Word document.  No units will be identified but if given permission, we will acknowledge respondents’ contribution. | Name of respondent  Name of hospital  Job role  Date survey completed  Please send any queries to |
| --- | --- |

|  |  | Doctors | ANNPs | Other professional |
| --- | --- | --- | --- | --- |
| 1. | Please estimate the proportion (%) of PICCs that are inserted by the staff groups above the boxes on the right. If no PICCs inserted in your unit tick here |  |  |  |
|  |  | **Yes** | **No** | **N/A – PICCs not inserted in our unit** |
| 2. | Do you use a care bundle (i.e. package of interventions designed to maximise asepsis and minimise infection complications) for **insertion** of PICCs? |  |  |  |
|  | If yes, approximately what month and year was this first introduced? |  | | |
| 3. | Do you use a care bundle (i.e. package of interventions designed to maximise asepsis and minimise infection complications) for **on-going care** of PICCs? |  |  |  |
|  | If yes, approximately what month and year was this first introduced? |  | | |
| 4. | Is compliance with insertion and on-going care bundles monitored and fed back to NICU staff to improve practice? |  |  |  |
| 5. | Do you routinely use a chlorhexidine-impregnated foam dressing (Biopatch) at the site of insertion? |  |  |  |
|  | If yes, what is usually the minimum gestation at which this is used? Please give your answer as weeks of gestation. |  |  |  |
| 6. | Do your unit guidelines mandate routine removal / replacement of a PICC after a specific period of time? |  |  |  |
|  | If yes, what is the usual time period in days? |  |  |  |
| 7. | Do your unit guidelines recommend PICC removal when a specific volume of feeds has been reached? |  |  |  |
|  | If yes, what volume (mls/kg/day)? |  |  |  |

|  | **8.** | **9.** | **10.** |
| --- | --- | --- | --- |
|  | What skin preparation do you use prior to insertion? | In your unit, prior to connection of parenteral nutrition, what is the area of the extension sets seen below in photographs A and B cleaned with, if anything? (Please number all that apply in order: 1=used most often) | |
|  | Please base your answer on your routine practice for a 29 week gestation baby weighing 900g.  If you have a 2-stage procedure, please select all that apply: 1 = used first. | 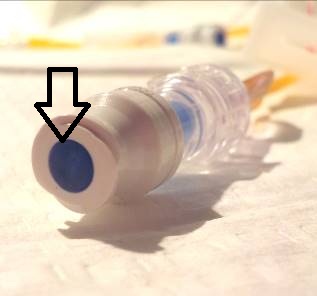  A | 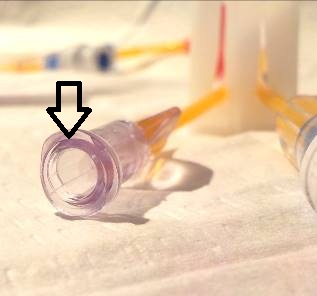  B |
| Nothing |  |  |  |
| 0.05% aqueous chlorhexidine |  |  |  |
| 0.5% aqueous chlorhexidine |  |  |  |
| 1% aqueous chlorhexidine |  |  |  |
| 2% aqueous chlorhexidine |  |  |  |
| 0.5% chlorhexidine in alcohol |  |  | ☐ |
| 1% chlorhexidine in alcohol |  |  |  |
| 2% chlorhexidine in alcohol |  |  |  |
| 70% isopropyl alcohol |  |  |  |
| Iodine preparation (aqueous or alcoholic) |  |  |  |
| We would only clean if became contaminated |  |  |  |
| This type of connection is not used |  |  |  |
| Specify if 'other' preparation used. |  |  |  |

| The PREVAIL team would like to acknowledge contributorship in any publications or outputs from this work. Please tick this box if you are happy for us to mention your name as a contributor. |  |
| --- | --- |

Please return the completed survey as a scan or photograph of both pages or a Word document and e-mail with subject title “PREVAIL Generalisability Survey – HOSPITAL NAME”

Alternatively, send by post to
